# The Neural Representation of Relational- and Collective-Self: Two Forms of Collectivism

**DOI:** 10.3389/fpsyg.2018.02624

**Published:** 2018-12-19

**Authors:** Yingcan Zheng, Zilun Xiao, Luqing Wei, Hong Chen

**Affiliations:** ^1^Faculty of Psychology, Southwest University, Chongqing, China; ^2^Department of Psychology, Army Medical University, Chongqing, China; ^3^School of Psychology, Jiangxi Normal University, Nanchang, China

**Keywords:** relational-self, collective-self, collectivism, relational collectivism, group collectivism, neural representation

## Abstract

The collectivism can be divided into two forms: relational collectivism and group collectivism. According to the cognitive representation of self, relational collectivism emphasizes the relational-self and group collectivism privileges the collective-self. However, it remains uncertain whether there is a difference between relational-self and collective-self under Chinese collectivism cultural. To address the above issue, the present study examined the neural representation of relational-self and collective-self during trait judgment tasks using functional magnetic resonance imaging (fMRI). The results showed that relational-self-reference compared with collective-self-reference generated stronger medial prefrontal cortex (MPFC) activity, indicating relational-self was more closeness and important in the self-concept than collective-self under East Asian cultural background. Relational-self and collective-self are unequally represented in the MPFC, providing direct neural evidence that the collectivism in China can be divided into relational collectivism and group collectivism.

## Introduction

There is considerable evidence that people have three cognitive representations of the self: the individual-self (cognitions related to one’s particularly traits, states and behaviors), the relational-self (cognitions related to relationships with close others), and the collective-self (cognitions related to membership groups) (Triandis, 1989; Brewer and Gardner, 1996). A series of experiments have provided support for the prominence of the individual-self compared with relational-self and collective-self. A meta-analysis indicated the primacy of the individual-self has been demonstrated across cultures (Gaertner et al., 2002). Sedikides et al. (2013) found that people have a stronger motive to seek individual-self verification than relational and collective selves. Investigators also showed that compared with relational and collective selves, imaging the loss of the individual-self elicited stronger emotion reactions (Gaertner et al., 2012). In addition, studies of participants in China provided evidence for the primacy of the private self (Huang et al., 2014; Zhu et al., 2016). Taken together, the abovementioned findings indicate that the primacy of the individual-self is a universal phenomenon in various cultural groups including China. However, whether there is a difference between relational-self and collective-self is not clear yet.

One of the central claims of cultural psychology is that an individual’s representations of self are influenced by individual’s culture of origin. The model of cultural difference in self-construal claims that East Asian cultures emphasize fundamental social connection with others and social group and encourage an interdependent view of a self. The interdependent self-construal model corresponds to the collectivism cultural descriptor (Markus and Kitayama, 1991). Furthermore, Brewer and Chen (2007) have divided the collectivism into two forms: relational collectivism and group collectivism. The theoretical distinction between two different types of collectivism are distinguished based on whether the social in-group (the target of collective orientation) is defined as a network of interpersonal relationships or as a depersonalized social category. According to the cognitive representation of self, relational collectivism emphasizes the relational-self and group collectivism privileges the collective-self (Mamat et al., 2014). However, there was few empirical evidences provided these two forms of collectivism under Chinese cultural. That is, is there a difference between relational self and collective self?

The neural basis of the self has been recognized as one of the most prominent problems in neuroscience (Northoff and Bermpohl, 2004; Northoff et al., 2006; Gillihan and Farah, 2005; Legrand and Ruby, 2009). Researchers used a self-referential task (Rogers et al., 1977) that requires judgment of whether a trait can describe the self or others to investigate the neural representation of self. Kelley et al. (2002) using functional magnetic resonance imaging (fMRI) found activation in the medial prefrontal cortex (MPFC) during judgments of personality traits of oneself versus a celebrity. As the MPFC activation is greater to trait words rated high versus low in self-relevance (Moran et al., 2006) and correlated with memory performances on recall of self-related trait words (Macrae et al., 2004; Ma and Han, 2011), it is therefore reasonable to infer that the MPFC is involved in encoding of self-relevance of stimuli (Northoff et al., 2006; Han and Northoff, 2009).

Similar to the individual-self, the information of relational and collective selves has high emotional significance, reward value, and/or more familiarity compared with other information (Chen et al., 2011; Tacikowski et al., 2014). Especially in China, the biases responses to our preferred stimuli can be extended from the individual-self to the relational-self and collective-self information, such as close other’s and the groups that we categorize ourselves as being members of (Sparks et al., 2016; Macrae et al., 2017; Truong et al., 2017). Our memory for relational-self (mother-name) is typically better than for material not related with relational-self (famous-name) (Fan et al., 2011), and brain activation was stronger during mother-judgments processing than famous-judgments processing, indicating the relational-self-reference effect (Zhu et al., 2007). Recent neuroimaging study with young adults has shown that processing information about oneself (individual-self) and family members (relational-self) shared common neural correlates. Zhu et al. (2007) examined whether trait judgments of both oneself and one’s mother activate the MPFC in Chinese university students. They found the MPFC activation in both contrasts of self- versus celebrity-judgments and mother- versus celebrity-judgments, and the contrast of self- versus mother-judgments did not show any significant activation. These results suggest that the MPFC underlies the neural underpinnings of oneself and one’s mother. The shared MPFC activity observed in the previous research may reflect the enhanced coding of self-relevance of both individual-self and relational-self.

Like the relational-self, the collective-reference effect was also confirmed in Chinese subjects. Yang and Huang (2007) found that recognition rates were significantly higher when trait words were encoded reference to Chinese relative to trait words processed in reference to American. The collective-self has shown to be able to facilitate memory as the individual-self and relational-self. Additionally, recognition rate and “remember” judgment rates were significantly lower in Chinese-reference processing than those in individual-self-reference processing, indicating that the effect of collective-self-reference on memory had a relatively smaller magnitude compared with individual-self. However, the neural mechanism of collective-self was not clear, and the present study aim to examine the MPFC activity during Chinese-reference processing. Moreover, few researches have compared the neural mechanism of relational-self and collective-self directly.

The goal of this study was to investigate the different neural mechanism of relational-self and collective-self in Chinese people. To achieve the above issue, we adopted a trait judgment task to assess the representation of relational-self and collective-self (Moran et al., 2006; Ma and Han, 2011; Han et al., 2016). Mother chose as relational-self, and Chinese chose as collective-self (Cai et al., 2012; Wang et al., 2017). Furthermore, the current study verify the special neural mechanism encoded the relational-self and explore the neural mechanism during collective-self-reference processing. Lu Xun as a famous person in China was chosen as the control condition of relational-self and American was chosen as the control condition of collective-self (Yang and Huang, 2007). We also applied a valence identification condition to control general perceptual and semantic processing factors and to evaluate the contribution of person knowledge and reference condition processing to the judgment tasks (Gao and Wang, 2017).

Accordingly, the present study was designed to examine the neural mechanism of collective-self and determined whether the brain activation of relational-self would be different compared with collective-self. If the brain activation patterns in relational-self-processing condition were different from collective-self-processing, this may indicate that the collectivism in China can be divided into relational collectivism and group collectivism. Based on these analyses, we hypothesized that the relation-self may activate stronger MPFC than collective-self during the trait-judgment processing. Understanding the complexities of relational collectivism and group collectivism and their interrelationships may prove to be critical to managing social change.

## Materials and Methods

### Participants

Prior to data collection, a power analysis was performed to determine the necessary number of subjects. Assuming a moderate effect size (η^2^ = 0.35), moderated power (1-β = 0.80), and a within factors repeated measures *F*-test, approximately 20 participants were required. Data collection ceased at the end of the academic term in which this minimum number was reached.

Thirty participants (after excluding four participants with excessive motion; fourteen men and sixteen women) (age: 18–25, mean 19.76 ± 1.42 years) were recruited from Southwest University of China. Participants were all right handed and reported no abnormal neurological or psychiatric history. All of them had normal or corrected-to-normal visual acuity. Informed consent was obtained in accordance with procedures and protocols that were approved by the human subjects review board of Southwest University in China. All methods were carried out in accordance with the approved guidelines.

### Stimuli and Procedure

**FIGURE 1:**
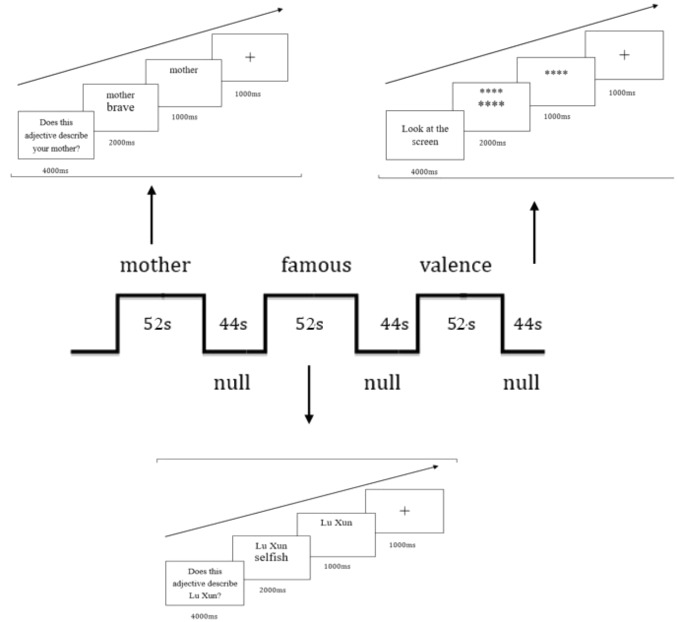
Illustration of stimuli and procedure during relational-self scans. Each scan consisted of three sessions of different judgment tasks. Two successive sessions were intervened by a null session of 44 s.

**FIGURE 2:**
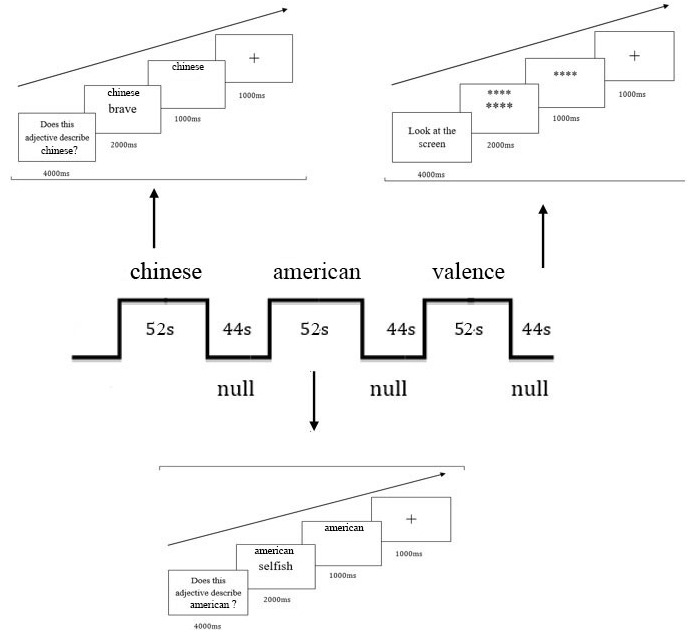
Illustration of stimuli and procedure during collective-self scans.

Participants were scanned during performing trait judgment tasks, and six functional runs were carried out for each subject. The stimuli were presented through an LCD projector onto a rear projection screen at the head end of the bore. The screen was viewed with an angled mirror positioned on the head coil. Three functional scans involved relational-self and the others involved collective-self. Each of the functional scans consisted of three judgment blocks. Three judgment tasks were conducted in each relational-self scan requiring subjects to judge if an adjective was proper to describe the mother, Xun Lu (a well-known Chinese writer) or to judge the valence of the words (positive or negative) (Figure 1). In addition, three judgment tasks were conducted in each collective-self scan requiring subjects to judge if an adjective was proper to describe Chinese, American, or a valence judgment (Figure 2). Each session lasted for 52 s began with a 4 s instruction that informed participants which judgment task was followed. Participants made judgments by pressing one of the two buttons using the left or right hand. The judgment tasks were intervened by null sessions during which subjects passively viewed two rows of asterisks (^∗^). Each null session lasted for 44 s. The order of the judgment tasks was randomly for each participant and the bottoms were counterbalanced between subjects.

Each trial in the judgment tasks consisted of a “cue” word (e.g., mother, other, Chinese, American, and valence) above a trait adjective presented for 2000 ms at the center of the screen. The trait adjective then disappeared while the ‘cue’ word stayed on the screen for 1000 ms, during which time participants made their responses. After each trail, the response was followed by a blank which was presented for 1 s before the next trial. Each of the Chinese characters in the cue word and trait words subtended a visual angle of 0.34° × 0.45° (width × height) and 0.85° × 1.14°, respectively at a viewing distance of 80 cm. Each symbol used in the null session subtended 0.23° × 0.23° (small ones) or 0.56° × 0.56° (large ones). All words and symbols were white on a black background.

A total of 240 unique adjectives consisting of two Chinese characters were selected from established personality trail adjective pools (Chinese words from Liu, 1990). The adjectives were classified into 6 lists of 40 words matched in word valence. Each list was pseudo-randomly selected for the trait judgment tasks in each scan, to ensure that the 6 lists of words randomly assigned to six scans among participants.

After scanning, participants completed the Self Construal Scale (SCS, adapted from Singelis, 1994) to measure their cultural orientations, and a measurement of the degree of closeness that they felt to their mother (adapted from Aron et al., 1992).

### fMRI Data Acquisition

Function imaging data was acquired using a Siemens TRIO 3T MRI scanner in the Key Laboratory of Cognition and Personality at the Southwest University in China. Participants lay supine with their heads snugly fixed with foam pads to minimize head movement and were instructed to keep still. A total of 150 BOLD Images were obtained using an Echo Planar Imaging (EPI) sequence with the following parameters: slices = 32; repetition time (TR) = 2 s; echo time = 30 ms; field of view (FOV) = 220 mm × 220 mm; flip angle = 90°; voxel size = 3.4 mm × 3.4 mm × 3 mm; thickness = 3 mm; matrix = 64^∗^64. A magnetization-prepared rapid gradient echo (MPRAGE) sequence was used to acquire high-resolution T1-weighted anatomical images.

### fMRI Data Analyses

DPARSF 2.3^1^ was used for data preprocessing analysis. Functional images were corrected for the acquisition delay between slices using the middle slice as the reference frame and further realigned to the first volume to correct for head movement. Participants with head motion exceeding ± 3.0 mm of translation or ± 3.0° of rotation were excluded from the dataset. Functional images were then normalized to EPI templates based on the Montreal Neurological Institute (MNI) space (resampling voxel size was 3 mm × 3 mm × 3 mm). The resulting normalized data were then spatially smoothed (8 mm FWHM Gaussian kernel) to increase signal to noise ratio.

Statistical analyses used a hierarchical random-effects model with two levels by SPM12 (the Welcome Trust Centre for Neuroimaging, London, United Kingdom). In the first-level analysis, the onsets and durations of each session were modeled using a General Linear Model (GLM) according to stimulus conditions. The movement correction parameters were added as covariates of no interest. All six conditions (mother-judgments, famous-judgments, valence-judgments during relational-self functional scans, Chinese-judgments, American-judgments, valence-judgments during collective-self functional scans) were included in the model. The contrast between trait judgments (mother/famous/Chinese/American) and matched valence judgments was defined in each subject. These individual contrast images were submitted to a second-level random-effect analysis. The significance threshold was set at *p* < 0.05 (Alphasim corrected, *K* > 54, individual voxel *p* < 0.001, determined by a 5000-iteration Monte-Carlo simulation; Slotnick et al., 2003). This threshold was also used for other exploratory whole-brain analyses.

A regions of-interest (ROI) was conducted to explore the representation difference of relational-self and collective-self in MPFC. ROI was defined as spheres centered at the peak voxels of MPFC activation in the contrast between relational-self and collective-self reference conditions, with radius of 6 mm using MarsBaR^2^. Contrast values were extracted from the ROIs by subtracting the coefficient estimates of the valence judgments condition from those of the trait judgment conditions. The fMRI signals were subjected to a repeated measure analysis of variance (ANOVA) with factors being Self (relational-self vs. collective-self) and Information type (self-relevant vs. non-self-relevant). Then the fMRI signals in the non-self-relevant condition were also subtracted from the self-relevant condition to index the relational-self and collective-self reference effects, respectively. A paired *t*-test was then conducted on the differential fMRI signals.

## Results

### Self-Report Measures

Results from the SCS showed that the participants were tended to be interdependent, self-construal compared with independent self-construal [*M* = 5.29, *SD* = 0.43 vs. *M* = 5.12, *SD* = 0.42, *t*(29) = 2.35, *p* < 0.05]. These results suggest collectivist cultural orientation among out Chinese sample. On the 7-point Likert scale of closeness between self and mother (1: no overlap; 7: fully overlap), the participants’ rating scores ranged from 2 to 7 (*M* = 4.38, *SD* = 1.41), suggesting the closeness between participants and their mothers was above the middle level, *t*(29) = 3.64, *p* = 0.001.

### Behavioral Results

**FIGURE 3:**
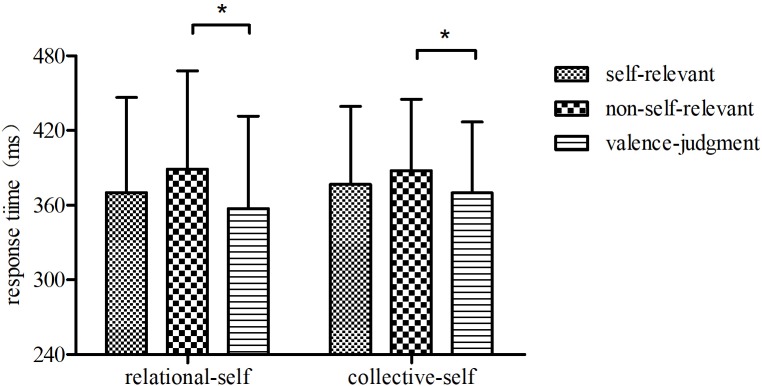
Response times during different judgment processing. Error bars denote standard errors. ^∗^*p* < 0.05.

Figure 3 shows the response times for each session type. ANOVA with factors being self-construal (relational-self vs. collective-self) and information type (self-relevant, non-self-relevant and valence) showed no significant main effect of self-construal, *F*(1,29) = 0.52, *p* > 0.05, and no significant interaction of self-construal and information type, *F*(1,29) = 0.42, *p* > 0.05. However, the main effect of information type was significant, *F*(2,28) = 6.64, *p* < 0.01, η^2^ = 0.32, *observed power* = 0.88. Bonferroni-corrected *post hoc* analyses showed that response latencies were significantly faster for valence-processing than for non-self-relevant information-processing, *p* < 0.01. However, the comparison of response times between self- and non-self-relevant information processing did not reach significant.

### fMRI Results

**FIGURE 4:**
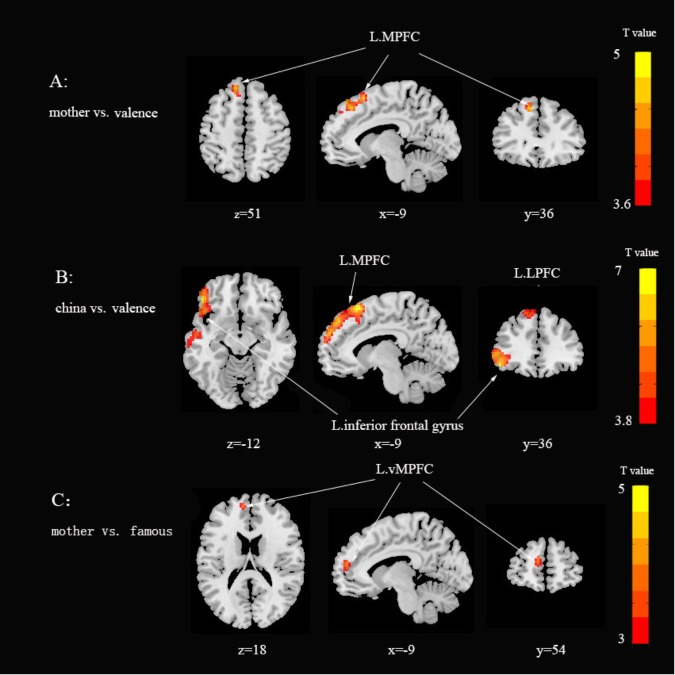
Brain activations reveals in different contrast, *p* < 0.05, corrected by Alphasim, a combined threshold of *p* < 0.001, and a minimum cluster size of 54 voxels. **(A)** mother vs. valence; **(B)** china vs. valence; **(C)** mother vs. famous.

To identify brain areas involved in the different encoding process, the whole brain analysis of mother-, famous-, Chinese-, and American-judgments were first contrasted with matched valence-judgment (Table 1 and Figures 4A,B). The results revealed activation in the left medial prefrontal cortex (MPFC, BA 8/9) for both the mother-, Chinese- and American- judgments, but not in the famous- judgment tasks. Chinese- and American- judgments also activated the inferior frontal gyrus (IFG, BA 47). This result showed that the MPFC was activated during both mother and Chinese-judgment processing, compared with valence-judgment.

**Table 1:** Regions of significant increased activation in comparison between mother, famous, China and America with valence judgments, main effect of self and interaction of self × information type (corrected, *p* < 0.05).

Condition/Regions	Voxel no.	BA	*X*	*Y*	*Z*	*t*-value
Mother minus valence						
L. MPFC	9	8	−9	36	51	4.70
Famous minus valence						
No significant activation area						
Chinese minus valence						
L. Inferior Frontal Gyrus	390	47	−48	36	−12	6.51
L. MPFC	179	9	−9	48	42	6.42
American minus valence						
L. Inferior Frontal Gyrus	408	47	−36	12	33	6.91
L. MPFC	253	8	−9	45	51	5.98
Mother minus famous						
L. MPFC	28	10	−9	54	18	4.26

*BA, Brodmann’s area; Voxel no., number of voxels in a cluster.*

To examine whether relational-self were differentially encoded in the MPFC relative to the collective-self, firstly whole brain analysis was calculated to contrast mother-judgments vs. Chinese-judgments. The contrast between mother-judgments and Chinese-judgments did not show any significant activation. Then the differences in brain activity during self-processing and non-self-processing were analyzed separately by paired *t*-test. The results found that ventral medial prefrontal cortox (vMPFC) showed increased activity linked to mother-judgments processing than famous-judgments, whereas the activations during Chinese-judgments and American-judgments processing were comparable (Figure 4C).

To assess the difference between relational-self reference and collective-self reference effects, we defined the neural substrates of the self-reference effect as increased neural activities associated with self-relevance- than non-self-relevance-judgments. The contrast between mother- and famous-judgments was used to index the relational-self reference effect, and the contrast between Chinese- and American- judgments to index the collective-self reference effect. The results of paired *t*-test showed that relational-self activated stronger MPFC (x/y/z = −9/57/9) than collective-self (10 contiguous voxels at *p* < 0.001 uncorrected).

**FIGURE 5:**
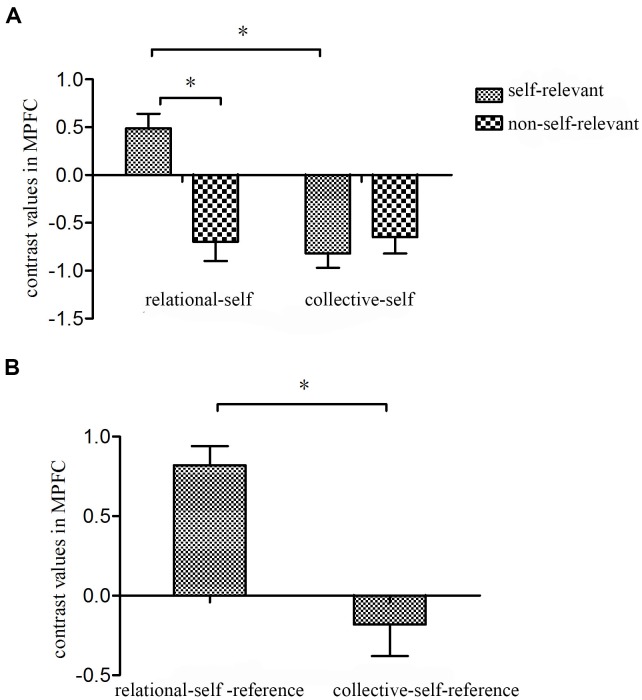
Represent fMRI signal changes in ROI in the self- and non-self-relevant information processing conditions for both relational- and collective-self. Error bars denote standard errors. **(A)** Represents fMRI signal changes in MPFC in the self-relevant and non-self-relevant conditions relative to valence condition; **(B)** Represents the differential fMRI signal changes in MPFC for relational-self- and collective-self-reference. ^∗^*p* < 0.05.

The ROI analysis calculated percent signal changes in the MPFC (centered at −9/57/9) relative to the matched valence judgments condition. 2 × 2 repeated ANOVA was conducted on the differential ROIs of the contrast values. The results confirmed that the main effect of self did not reach significant. However the main effect of Information Type was significant, *F*(1,29) = 6.83, *p* = 0.01, η^2^ = 0.17, *observed power* = 0.71. The interaction of self and information type was also significant, *F*(1,29) = 13.06, *p* = 0.001, η^2^ = 0.28, *observed power* = 0.94. Simple effect analysis revealed that signals in MPFC was greater to mother- than famous- and Chinese-judgment, both *p* = 0.001, but did not differ between Chinese- and American-judgments, *p* = 0.41 (Figure 5A). Then a paired *t*-test was conducted on the fMRI signals of relational-self and collective-self reference effects, and the results confirmed that MPFC activated larger during relational-self than collective-self-referential processing, *t*(29) = 3.27, *p* < 0.01(Bonferroni-corrected, Figure 5B).

To examine whether the MPFC activity associated with the relational- and collective-self can predict subjective evaluations of one’s self-construal and intimacy with mother, we calculated correlations between subjective rating scores and MPFC activity from the ROI defined in the contrast in association with relational- and collective-self reference. No significant correlation was found.

## Discussion

Using fMRI, we examined the neural mechanism during the collective and relational-self–reference processing. The results showed greater activation in the MPFC during mother- and Chinese-judgment processing compared to valence processing. Furthermore, there is a stronger activity of MPFC during mother-judgment compared with Chinese-judgment. Moreover, after subtracting the non-self-relevant information judgments, the relational-self-reference showed greater MPFC activation than collective-self. These results provided first evidence for greater brain activation in MPFC of relational-self compared with collective-self, suggesting that relational-self was more closeness and important in the self-concept than collective-self under East Asian cultural background.

The aim of the current study was to compare the hierarchical differences during cognitive processing between relational-self and collective-self. ROI analysis revealed that Chinese-judgments processing exhibited smaller activation of MPFC than mother-judgments processing. Consistent with previous motivation researches (Huang et al., 2014; Mamat et al., 2014), the results found that participants ranked relational-self and individual-self similarly, and both were more important than collective-self in both individualistic and collectivistic cultures. The relational-self, as a representation of interpersonal networks, is more individualized and personally specific than the collective-self, and therefore has a higher emotional significance for individuals (Brewer and Chen, 2007). During relational-self processing, Chinese people use more attentional resources to identify and evaluate relevant memories and emotional information about close people. Based on the previous researches, we may infer that MPFC contributes to the encoding of self-relevance of stimuli (Kelley et al., 2002; Zhu et al., 2007), and the MPFC also mediates a function to represent difference self-relatedness information (Wang et al., 2011). Self which closer to the center of self-concept, the stronger MPFC will be activated. Nehrlich et al. (2018) proposed to explain the hierarchical of self-construal using different self roles, and they found that the collective-self lagged somewhat behind the individual and relational selves, being less functional in reaching one’s teleological ideal. Consistently, relational-self-reference effect activated stronger MPFC than that of collective-self.

Moreover, the activation of the relational- and collective-self was more obvious on the left hemisphere, whereas considerable research indicated that the right hemisphere played a crucial role in self-referential processing (Han and Northoff, 2008, 2009). However, the self-concept mentioned in previous studies was more related to individual-self and failed to consider the social attribute of self. Relational-self would involve the relationship with others and contains the concept of others, and collective-self would incorporate national symbolism and social identity. The left lateralization for relational- and collective-self found in the present study was consistent with previous studies by showing that the collective-self-referential effect was more obvious on the left electrode sites (Zhao et al., 2009).

The fMRI results showed the MPFC was activated both during mother-, and Chinese-judgments processing compared with valence-judgment processing. A large body of research on the self-reference has established that MPFC is associated with self-related processing and coded self-related information, including self-reflection on personality traits, self- face recognition, and self-related information retrieval (Zhu et al., 2007; Moran et al., 2009; Pfeifer et al., 2013). Therefore, as the main brain region of self-representation, the MPFC activation provides neural underpinnings for the self-concept consisted of relational-self and collective-self under East Asian cultural background. In addition, compared with valence-judgment processing, Chinese-judgments and American-judgments both induced stronger IFG. These brain areas were involved in semantic processing and motor sensation (Sharot et al., 2012), suggesting that during Chinese and American-judgments processing participants performed more semantic processing.

Consistent with previous researches, mother-judgment activated stronger vMPFC than famous-judgment processing. Studies of self-reference processing provided evidence that vMPFC was primarily responsible for tagging self-related information, and involving in the automated social cognitive processing of experienced areas (Lieberman et al., 2004; Northoff et al., 2006; Van der Meer et al., 2010; Jenkins and Mitchell, 2011). Compared with famous-judgments processing, the stronger activation in vMPFC during mother-judgments indicated that the evaluation of personality traits about mother had been incorporated in the self-domain. One’s mother as relational-self, is part of self-concept, and the judgment of mother was an automate process that does not require effort. Nevertheless, as collective-self, Chinese-judgments did not generate a special brain mechanism compared with American-judgments. Chinese-judgments activated similar dorsal medial prefrontal cortex (dMPFC) compared with American-judgments. Compared to vMPFC activation during mother-judgments, dMPFC was mainly involved in the evaluation of self-related information from other’s perspective (Moran et al., 2009; Van der Meer et al., 2010). Enhanced activation in the dMPFC occurred in goal-oriented tasks such as social cognition and propositional thinking that required conscious effort (Jenkins and Mitchell, 2011). Participants needed more conscious effort to compare and evaluate themselves according to Chinese and American perspectives. An ERP study using odd ball task also revealed that P2, N2, and P3 component during Chinese and American conditions and showed no significant difference (Chen et al., 2011). This may indicate that the sense of self perception formed by collective-reference was weaker, and Chinese and American were not different enough to induce differences (i.e., floor effect).

One limitation of our study is that our sample consisted solely of Chinese participants with interdependent self-construal. Given that the overlapping neural representation between the self and mother is stronger in Chinese interdependent self than in Westerners independent self (Zhu et al., 2007), it is possible that the MPFC activation of the relational-self and collective-self observed in this work might not generalize to people from more independent cultural contexts. Future research needs to explore whether culture plays a modulatory role in the neural representation of relational-self and collective-self. Moreover, we only selected countries as a representative of the collective-self. Previous ERP researches with Chinese people also used one’s province, old school, and nation (Zhao et al., 2009, 2011; Chen et al., 2011; Liu et al., 2015; Wang and Hamilton, 2015) as the collective-self. Future research needs to explore whether the different groups activate brain areas similar to one’s country.

## Conclusion

The current study compared the processes engage and neural mechanism during the relational-self- and collective-self-reference. The direct comparison of relational- and collective-self enriched the study of the cognitive hierarchy of self in Chinese brain. The MPFC activation was stronger during relational-self compared with collective-self reference, indicating that the processing of relational-self involves more attentional resources to identify and evaluate relevant memories and emotional information, and confirming that the Chinese people emphasis more relational collectivism than group collectivism. In addition, the left lateralization for relational- and collective-self indicated that the influence of social situation on self was mainly represented in the left brain. The different brain activation of relational- and collective-self confirmed that the collectivism in China can be divided into relational collectivism and group collectivism, and different social connection such as interpersonal relationship and depersonalized social category have distinct effect on one’s self.

## Open Access

This article is distributed under the terms of the Creative Commons Attribution 4.0 International License (http://creativecommons.org/licenses/by/4.0/), which permits unrestricted use, distribution, and reproduction in any medium, provided that appropriate credit is attributed to the original author(s) and the source provides a link to the Creative Commons license and indicates whether changes were made.

## Ethics Statement

All procedures performed in studies involving human participants were in accordance with the ethical standards of the institutional and/or national research committee and with the 1964 Helsinki declaration and its later amendments or comparable ethical standards. Informed consent was obtained from all individual participants included in the study.

## Author Contributions

YZ, ZX, and HC conceived and designed the experiments. YZ and ZX performed the data analysis and wrote the manuscript. LW and HC reviewed the manuscript.

## Conflict of Interest Statement

The authors declare that the research was conducted in the absence of any commercial or financial relationships that could be construed as a potential conflict of interest.

## Footnotes

1 http://rfmri.org/DPARSF

2 http://marsbar.sourceforge.net
